# Complete chloroplast genome sequence of *Rosa roxburghii* and its phylogenetic analysis

**DOI:** 10.1080/23802359.2018.1431074

**Published:** 2018-02-01

**Authors:** Qian Wang, Huan Hu, Jiaxing An, Guohui Bai, Qunli Ren, Jianguo Liu

**Affiliations:** a Research Center for Medicine & Biology, Zunyi Medical University, Zunyi, China;; b Department of Gastroenterology, Affiliated Hospital, Zunyi Medical University, Zunyi, China

**Keywords:** *Rosa roxburghii*, Rosaceae, chloroplast genome, phylogenetic analysis, Chinese traditional medicine

## Abstract

*Rosa roxburghii* Tratt. is a famous Chinese traditional medicine with long history, here we characterized the whole chloroplast (cp) genome sequence of *R. roxburghii* by Illumina pair-end sequencing. The complete cp genome was 156,749 bp in length, containing a large single copy (LSC) region of 85,862 bp and a small single copy (SSC) region of 18,791 bp, which were separated by a pair of 26,053 bp inverted repeat regions (IRs). The genome contained 139 genes, including 88 protein-coding genes (82 PCG species), 39 tRNA genes (32 tRNA species), and eight ribosomal RNA genes (four rRNA species). Most of the gene species occur as a single copy, while 17 gene species occur in double copies. The overall AT content of *R. roxburghii* cp genome is 62.8%, while the corresponding values of the LSC, SSC, and IR regions are 64.8, 68.7, and 57.4%, respectively. Further, phylogenetic analysis suggested that *R. roxburghii* is closely related to *R. odorata* var. *gigantea*.

## Introduction

*Rosa roxburghii* Tratt. is a wild perennial shrubs of Rosaceae that originates from Southwestern China. For hundreds of years, *R. roxburghii* has been used as traditional medicine in China. With the development of modern medical research, fruit extracts of this plant are believed to have numerous beneficial actions on arteriosclerosis, cancer, immunity, and stress (Hu et al. 1994; Wang and Xia 1996). The therapeutic effect of *R. roxburghii* fruit extracts could be a result of a combination of various compounds that contain ascorbic acid, polyphenols, and superoxide dismutase (Wu et al. 1994; Zhang et al. 2001). Besides study on the effective components and action mechanism, a good knowledge of genetics information would contribute to its development and utilization strategy. In this study, we assembled and characterized the complete chloroplast (cp) genome sequence of *R. roxburghii* based on the Illumina pair-end sequencing data.

Sample of *R. roxburghii* was collected from Meitan (Guizhou, China; 107°28′15.01″ E, 27°46′51.41″ N), and maintained in Research Center for Medicine & Biology of Zunyi Medical University. Genomic DNA was extracted following the modified CTAB method (Doyle and Doyle 1987). The whole-genome sequencing was conducted with 150 bp pair-end reads on the Illumina Hiseq Platform (Illumina, San Diego, CA). About 8 million reads were obtained and kept by mapping to cp genomes of *Rosa odorata* var. *gigantea* (KF753637) (Yang et al. 2014) using BWA (Li and Durbin 2009) and SAMtools (Li et al. 2009), then assembled using Velvet (Zerbino and Birney 2008). Annotation was performed with Plann (Huang and Cronk 2015). The complete cp genome sequence together with gene annotations was submitted to GenBank under the accession numbers of KX768420 for *R. roxburghii*.

The complete cp genome of *R. roxburghii* is a double stranded, circular DNA 156,749 bp in length, which contains two inverted repeat (IR) regions of 26,053 bp each separated by a large single copy (LSC) and a small single copy (SSC) region of 85,862 and 18,791 bp, respectively. The genome contained 139 genes, including 88 protein-coding genes (82 PCG species), 39 tRNA genes (32 tRNA species), and eight ribosomal RNA genes (four rRNA species). Most of the gene species occur as a single copy, while 17 gene species occur in double copies, including all rRNA species, seven tRNA species and six PCG species. The overall AT content of *R. roxburghii* cpDNA is 62.8%, while the corresponding values of the LSC, SSC, and IR regions are 64.8, 68.7, and 57.4%, respectively.

**Figure 1.**
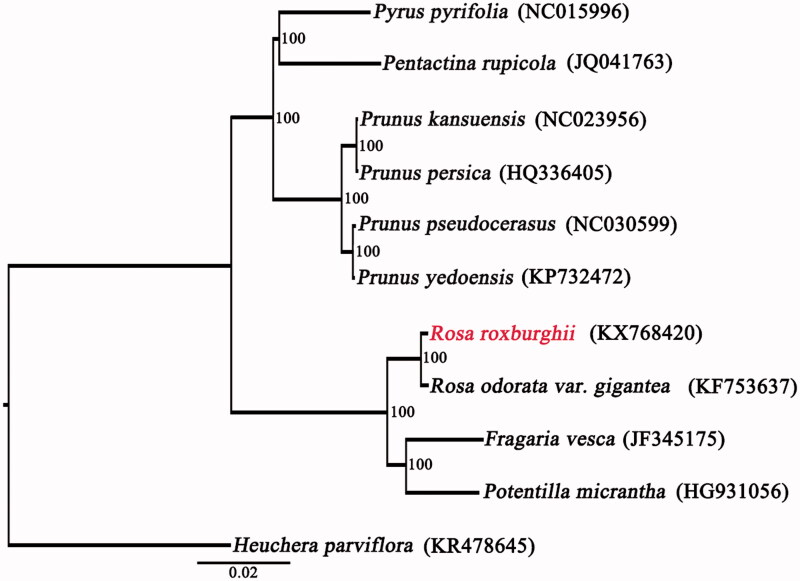
Maximum likelihood (ML) tree was constructed with other nine CP genome sequences of Rosaceae, *Heuchera parviflora* (KR478645) was used as the outgroup. Bootstrap support values (%) are indicated in each node. GenBank accession numbers: *Prunus kansuensis* (NC023956), *P. persica* (HQ336405), *P. pseudocerasus* (NC030599), *P. yedoensis* (KP732472), *Pyrus pyrifolia* (NC015996), *Pentactina rupicola* (JQ041763), *Potentilla micrantha* (HG931056), *Fragaria vesca* (JF345175), and *Rosa odorata* var. *gigantea* (KF753637).

To validate the phylogenetic position of *R. roxburghii*, a maximum likelihood (ML) tree with 100 bootstrap replicates was inferred using RaxML version 8 (Stamatakis 2014) containing plastid genomes of other nine Rosaceae species, *Heuchera parviflora* was used as the outgroup. The phylogenetic position of *R. roxburghii* is closely related to *R. odorata* var. *gigantea* (Figure 1). This complete cp genome can be subsequently used for population, phylogenetic and cp genetic engineering studies of *R. roxburghii*, and such information would be fundamental to formulate potential development and management strategies for this important medicine plant.
